# In‐situ extraction and impregnation of black walnut husk into polyethylene film using supercritical carbon dioxide with an ethanol modifier

**DOI:** 10.1002/fsn3.1348

**Published:** 2019-12-17

**Authors:** Jonathan E. Wenzel, Veronica Moorman, Lihua Wang, Isaiah Spencer‐Williams, Mitchell Hall, Cheryl S. Samaniego, Michelle L. Ammerman

**Affiliations:** ^1^ Kettering University Flint MI USA

**Keywords:** antioxidants, LDPE, supercritical fluid extraction, supercritical fluid impregnation, walnut husks

## Abstract

Walnuts are commonly cultivated for their kernel, which is a rich source of antioxidant phenolic compounds. The husk likewise contains antioxidant and antimicrobial compounds, but is typically discarded without further processing. Antioxidant compounds are useful in creating active packaging films, but typically decompose at melt extrusion temperatures in polymer processing. Due to carbon dioxide's low critical point and ability to swell polymer films, supercritical carbon dioxide may be used to impregnate phenolic compounds into polymers. For this study, a novel technique is used to simultaneously produce walnut husk extracts and impregnate the extract into polymer films in the same batch extractor using supercritical carbon dioxide with a 15 wt‐% ethanol modifier at 60°C at 19.4 MPa. The effect of varying the loading of walnut husk in the extractor upon impregnation mass was evaluated with the impregnation mass of the film increasing with walnut husk loading. It was determined by FTIR, as well as the reduction of the protein cytochrome *c*, that antioxidant compounds may be extracted from walnut husks and impregnated into low‐density polyethylene film (LDPE) by this technique.

## Graphical abstract

Supercritical carbon dioxide with a 15 wt‐% ethanol modifier was used in an original technique to simultaneously produce walnut husk extracts and impregnate the extract into low density polyethylene films in the same batch extractor. It was determined that the impregnation mass of the film increases with walnut husk loading. It was determined by FTIR, as well as the reduction of the protein cytochrome c, that antioxidant compounds from walnut husks were impregnated into the film.

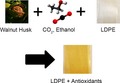

## 1 INTRODUCTION

In food processing, quite frequently the part of the plant that is discarded or composted after processing contains a rich variety of beneficial phytochemicals in comparison with the edible part of the plant (Guo et al., 2003). In many berry plants, peels, stems, and seeds are discarded and contain a higher amount of antioxidant and antioxidant compounds compared with the edible pulp (Guo et al., 2003). For walnuts specifically, the kernel and shells are commonly used, yet the husk is discarded. Walnut husks, stems, leafs, shells, and kernels all exhibit antioxidant potential (Wang et al., 2015; Yaylaci, Kolayli, Kucuk, Karaoglu, & Ulusoy, 2007). Phenolic compounds present in walnut husks and walnut extracts show numerous beneficial properties including antioxidant activity, anti‐inflammatory activity, antimicrobial properties, and cytotoxic effects against melanoma cells (Aithal, Kumar, Rao, Udupa, & Rao, 2009; Fernandez‐Agullo et al., 2013; Mikulic‐Petkovsek, Slatnar, Veberic, Stampar, & Solar, 2011; Oliveira et al., 2008; Tabaraki & Rastgoo, 2014; Wang et al., 2015; Wenzel et al., 2016). Since walnut husks are commonly discarded, despite being a source of antioxidants, walnut husks present a potential for valorization.

There is growing negative consumer sentiment toward processed foods and foods with added preservatives resulting in a demand for minimally handled organic produce and naturally derived products. The challenge therein lies in stabilizing food without artificial preservatives from the point of harvest, or production, through distribution to market. A potential means of protecting food from decay and diminished flavor without preservatives is to use packaging impregnated with naturally derived antioxidant compounds. Indeed, this is an active area of research. For example, Ecoflex® impregnated with olive leaf extract, linear low‐density polyethylene (LLDPE) impregnated with eugenol, and PET/PP impregnated with mango leaf extract all exhibit antioxidant potential (Belizon, Fernandez‐Ponce, Casas, Mantell, & de la Ossa‐Fernandez, 2018; Goni, Ganan, Strumia, & Martini, 2016; Marcos et al., 2014). Many naturally derived compounds and extracts, such as cinnamaldehyde, mango leaf, and clove essential oil, are advantageous in producing films that exhibit antimicrobial properties (Belizon et al., 2018; Mulla et al., 2017; Villegas et al., 2017). Active food packaging produced with plant extracts and antioxidant compounds has demonstrated an increased shelf life for produce and poultry (Belizon et al., 2018; Mulla et al., 2017). The impact will be food brought to market with minimal artificial preservatives, increased shelf life, and decreased waste.

There are a variety of methods and solvents that may be used for extraction that result in different recovery of polyphenolic compounds from agricultural process residues (Akay, Alpak, & Yesil‐Celiktas, 2011; de Campos, Leimann, Pedrosa, & Ferreira, 2008; Fontana, Antoniolli, & Bottini, 2013; Laroze, Diaz‐Reinoso, Moure, Zuniga, & Dominguez, 2010). Given the low polarity of phenolic compounds present in agricultural process residues, such as walnut husks, slightly polar solvents are preferable for extraction. Extraction of a variety of agricultural process residues such as walnut husks and grape pomace at subcritical conditions are commonly performed using ethyl acetate, ethanol, methanol, or mixtures of these solvents with water (Fontana et al., 2013; Lapornik, Prosek, & Wondra, 2005). Downstream applications must also be considered in solvent selection, importantly extracts prepared for ingestion, skin application, or food preparation require solvents that are generally recognized as safe (GRAS) by the United States Food and Drug Administration, such as carbon dioxide and ethanol. Supercritical carbon dioxide and ethanol are both nontoxic solvents with excellent solvating properties. Fluids above both the critical temperature and pressure are supercritical fluids and exhibit both gas‐ and liquid‐like properties. Supercritical carbon dioxide is a relatively nonpolar solvent, with a critical point of 31.1°C, that can be used to process temperature‐sensitive compounds and substrates such as extraction of hops, flavors, and essences (DeSimone, 2002). Given that supercritical carbon dioxide is relatively nonpolar at moderate pressures, it will result in lower selectivity as a solvent toward extracting polyphenolic antioxidant compounds (Leeke et al., 2005). To facilitate extraction of phenolic compounds with supercritical carbon dioxide, ethanol is commonly added as a modifier to increase the polarity of the mixture, which can be effective for extracting antioxidants from a variety of agricultural process residues such as grape pomace and walnut husks (Casas et al., 2010; Oliveira et al., 2013; Pinelo et al., 2007; Wenzel et al., 2016).

In addition to acting as a solvent, supercritical carbon dioxide can absorb and desorb from polymers such as polyethylene, as well as swell and relax polymer matrices while at supercritical fluid conditions, thereby enabling rapid diffusion of chemicals into polymers (Boyer, Klopffer, Martin, & Grolier, 2007; Chaudhary & Johns, 1998; Cooper & DeSimone, 1996; Li & Han, 2000; Lopez‐Gonzalez, Compan, & Riande, 2007). Provided supercritical carbon dioxide's low critical temperature, high diffusivity, and ability to swell polymers, it may be used to impregnate polymers with thermally sensitive compounds, such as pharmaceuticals (Lopez‐Periago et al., 2008; Potter et al., 2015; Uzer, Akman, & Hortacsu, 2006). Likewise, supercritical carbon dioxide has been demonstrated to impregnate chemical compounds such as 2‐nonanone, eugenol, and thymol to create antioxidant and antimicrobial active packaging (Goni et al., 2016; Mir et al., 2017; Rojas et al., 2015). Additionally, supercritical carbon dioxide, in both batch and semicontinuous modes of operation, was demonstrated to impregnate polyphenols from mango and olive leaf extracts into polymer films, as evidenced by the films demonstration of antioxidant potential (Cejudo Bastante et al., 2019; Villegas et al., 2017).

While pure chemicals, chemical mixtures, and plant extracts have been reported to be impregnated into polymer films, the feasibility of simultaneously extracting and impregnating antioxidant compounds from plant matter into polymers has not been reported. Since supercritical fluid mixtures are used both for extraction and for impregnation, it is conceivable that extraction and impregnation may take place in‐situ within the same high‐pressure vessel, or extractor, thereby eliminating a potential production process step. Additionally, walnut husk extracts do present an opportunity to be impregnated into films to produce antifungal and antioxidant packaging (Latos & Masek, 2019). The ability of supercritical carbon dioxide with an ethanol modifier to simultaneously extract antioxidants from green walnut husk into low‐density polyethylene films (LDPE) was evaluated, and the films tested for change in composition and antioxidant potential.

## 2 MATERIALS AND METHODS

### 2.1 Chemicals

The following reagent grade or purer chemicals were used: molecular biology grade ethanol (200 proof) and hydrochloric acid from Fisher Scientific; equine cytochrome *c* and quercetin from Sigma‐Aldrich; tris(hydroxymethyl)aminomethane base from Dot Scientific. Nitrogen (99.998%) and carbon dioxide (99.5%) were from Praxair. All chemicals were used without further purification. A total of 0.15 mm thick LDPE was from McMaster‐Carr.

### 2.2 Plant material

Black walnut trees, *Juglans nigra*, are native to North America. Walnuts used in this study were retrieved in southeastern Michigan following abscission while the husks were green at the conclusion of the 2017 growing season. The husks were manually removed shortly after retrieval. To aid in grinding, the husks were first frozen in a nitrogen‐purged container at −4°C, then ground frozen using a coffee grinder to a particle size between 20 and 50 mesh. Immediately following grinding, the husks were stored in double‐sealed nitrogen‐purged container in the dark at −4°C until time of use. The moisture content of the walnut husks was approximately 70% water by weight.

### 2.3 Extraction/impregnation system

**Figure 1:**
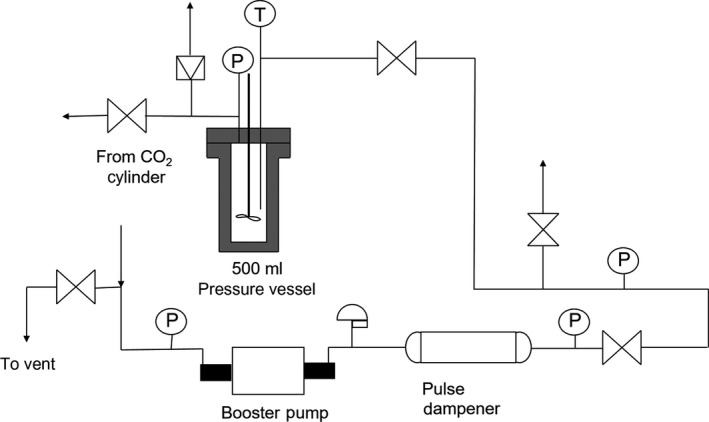
Process flow diagram for the stirred extraction/impregnation system modified with gas delivery system

Simultaneous extractions and impregnations were performed using a custom‐built batch extraction system, Figure 1. The system consisted of a 500 ml Hastelloy C 276® agitated high‐pressure vessel with thermowell and electric heating mantle (Parr Instruments, model 4575B), and a pneumatically driven, two‐stage gas booster pump (Haskel International, model 80875‐DP‐28881). A 1 L double‐ended sample cylinder (Swagelok, 316L‐50DF8‐10) was used as a pulse dampener. All shut‐off valves were two‐way straight valves with a regulated stem (Autoclave Engineers, part number SW4081). Pressure was indicated by a gauge as well as a pressure transducer with indicator. The extraction temperature was measured using a type J thermocouple with indicator. All tubing and fittings were 316 stainless steel.

### 2.4 Simultaneous extraction/impregnation method

For the simultaneous extraction and impregnation of walnut husks, predetermined amounts of walnut husk and ethanol were weighed and placed into the clean 500 ml pressure vessel. The experimental conditions were chosen to match the center point of a factorial design of experiments of a previous study evaluating the effects of processing conditions upon antioxidant potential of walnut husk extract (Wenzel et al., 2016). The amount of walnut husk was varied from 0:1 to 1:1 walnut husk to ethanol by mass. The amount of ethanol added to the pressure vessel was such that the target solvent concentration was 15 wt‐% ethanol with the balance being carbon dioxide at 60°C and 19.4 MPa. Additionally, a 3.9 × 7.1 cm piece of LDPE with a thickness of 0.15 mm was weighed and suspended in the pressure vessel. The pressure vessel was sealed, heated to 60°C, and then pressurized to 19.4 MPa with carbon dioxide. The temperature was then held constant at 60°C for 60 min. During heating and the 60 min hold time, the pressure vessel was agitated at a rate of 500 rpm. Next, without agitation, the extraction system was allowed to cool, gradually depressurized, opened, and the extract in liquid ethanol and polymer film retrieved. The film was rinsed with ethanol, dried, and weighed. Both the extract and the film were stored in separate, sealed, nitrogen‐purged containers in the dark at 4°C to protect from degradation until further analysis. For comparison purposes, identical impregnation conditions of 15 wt‐% ethanol with the balance being carbon dioxide at 60°C and 19.4 MPa with an impregnation time of 60 min under agitation were used to impregnate quercetin into LDPE film as a control with a loading of 2.021 g quercetin to 79.011 g ethanol (0.0256 quercetin/ethanol). Quercetin was chosen since it is known to be soluble in supercritical carbon dioxide with ethanol (Chafer, Fornari, Berna, & Stateva, 2004).

### 2.5 Fourier transformed infrared spectrometric analysis (FTIR)

The infrared spectra of the LDPE films before and after impregnating with walnut husk extracts were taken at room temperature using an Attenuated Total Reflectance (ATR) accessory on a Nicolet™ is50 Fourier Transform Infrared (FTIR) spectrophotometer (Thermo Scientific™). Each spectrum was an average of 16 scans in the range of 400–4,000 cm^−1^ with a resolution of 4 cm^−1^.

### 2.6 Biological relevance testing

Six mm diameter circles of treated and untreated films were punched out for biological testing. Each punched disk was placed in 350 μl of 0.3 M tris (hydroxymethyl) aminomethane buffer titrated to pH 7.4 with hydrochloric acid (with or without 5 μM oxidized cytochrome *c*) in a 96‐well plate with each condition replicated in quadruplicate. Blank, cytochrome *c* (5 μM), quercetin (1, 0.2, 0.04, 0.008 μg/ml), and a combined control (yielding reduced cytochrome *c*) were also run in quadruplicate on the same plate. Samples were incubated for 3 hr at room temperature, with lateral shaking every hour. Subsequently, 300 μl was taken out of each well and placed in a clean, UV‐vis transparent polystyrene 96‐well plate (Dot Scientific). UV‐vis spectra (350–750 nm with a step size of 1 nm) were taken using a SpectraMax M2 Multiplate Reader running SoftMaxPro 7.0.3 (Molecular Devices). Arithmetic means and standard deviations were calculated for each condition at each wavelength.

## 3 RESULTS AND DISCUSSION

### 3.1 In‐situ supercritical carbon dioxide and ethanol extraction and impregnation

**Figure 2:**
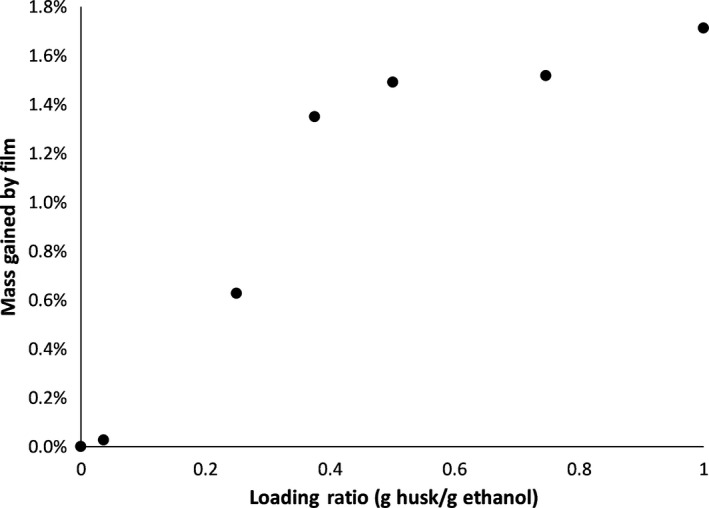
Percentage increase of LDPE film mass as related to loading ratio of walnut husk to ethanol for in‐situ extraction of walnut husk and impregnation into polymer film using supercritical carbon dioxide with an ethanol modifier

The effects of simultaneous extraction of ground, green walnut husks of the black walnut tree, *J. nigra*, and impregnation into LDPE using supercritical carbon dioxide with a 15 wt‐% ethanol modifier at 60°C in an agitated high pressure vessel for 60 min at 19.4 MPa were evaluated, which is the center point of the factorial design experiment from our previous study (Wenzel et al., 2016). The supercritical fluid extraction of black walnut husk using supercritical carbon dioxide with an ethanol modifier was previously evaluated at identical conditions with a loading of 0.14 g walnut husk per gram ethanol. At these conditions, extracts were shown to exhibit antioxidant potential of 0.0554 mmol trolox equivalent/g of walnut using the ferric reducing ability of plasma (FRAP) assay and 7.13 mg gallic acid equivalent/g of walnut using the total phenolic content (TPC) assay (Wenzel et al., 2016). For extraction and impregnation, the mass loading of walnut husk with respect to ethanol content was varied from 0 to 1 g of walnut husk per gram of ethanol. The treated film was evaluated for changes in mass, visually, by FTIR, and antioxidant potential assays. The film was weighed prior to and following extraction and impregnation. The green walnut husks were extracted without drying as a pretreatment since previous studies have shown that drying may decrease antioxidant potential such as *Mentha balsamea* (peppermint), *Echinacea angustifolia* (narrow‐leaves purple coneflower), and *J. nigra* (black walnut) (Maggini et al., 2010; Wenzel et al., 2016; Yi & Wetzstein, 2011). It was found that as the walnut husk loading was increased in the pressure vessel, the final film mass also increased, with a loading of 0.0361 husk/ethanol resulting in a negligible increase in mass and a mass loading of 1.00 husk/ethanol reaching a maximum of 1.712% mass increase in polymer, Table 1. This is to be expected; as the amount of walnut husk increased, the amount and availability of chemicals present to be extracted should increase. Indeed, in ultrasound‐assisted extraction of chokeberries, the greater the solid to solvent ratio, the higher the antioxidant potential of the extract (d'Alessandro, Kriaa, Nikov, & Dimitrov, 2012). However, as the walnut husk to ethanol mass loading approaches 0.5 husk/ethanol, the rate of increase of the polymer film begins to plateau, Figure 2. This may be indicative that the solvent system is becoming saturated with walnut husk extract at a 0.5 husk/ethanol mass ratio, which could provide direction toward optimizing the process in the future. In comparison, a film exposed to supercritical carbon dioxide with 15 wt‐% ethanol, but no walnut husk, showed no corresponding increase in mass. This indicated that the mass gained by the film was due to chemicals extracted and impregnated from the walnut husk, not due to a reaction between the film and the extraction solvent.

**Table 1:** Changes in LDPE film mass as a function of walnut husk loading using supercritical carbon dioxide with an ethanol modifier

Pressure vessel loading			Low‐density polyethylene film			
Husk (g)	Ethanol (g)	Husk/ethanol	Initial mass	Final mass	Mass gained	% Mass gained
0	79.504	0	0.3710	0.3710	0	0
2.8505	79.002	0.0361	0.3869	0.3870	0.0001	0.026%
19.769	79.041	0.2501	0.3990	0.4015	0.0025	0.627%
29.651	79.042	0.3751	0.4079	0.4134	0.0055	1.348%
39.531	79.026	0.5002	0.4161	0.4223	0.0062	1.490%
59.032	79.032	0.7469	0.4086	0.4148	0.0062	1.517%
79.015	79.017	0.9999	0.4146	0.4217	0.0071	1.712%

Note

Extraction/impregnation was conducted at 19.4 MPa, 60°C, 80 wt‐% carbon dioxide, 15 wt‐% ethanol, for 60 min.

**Figure 3:**
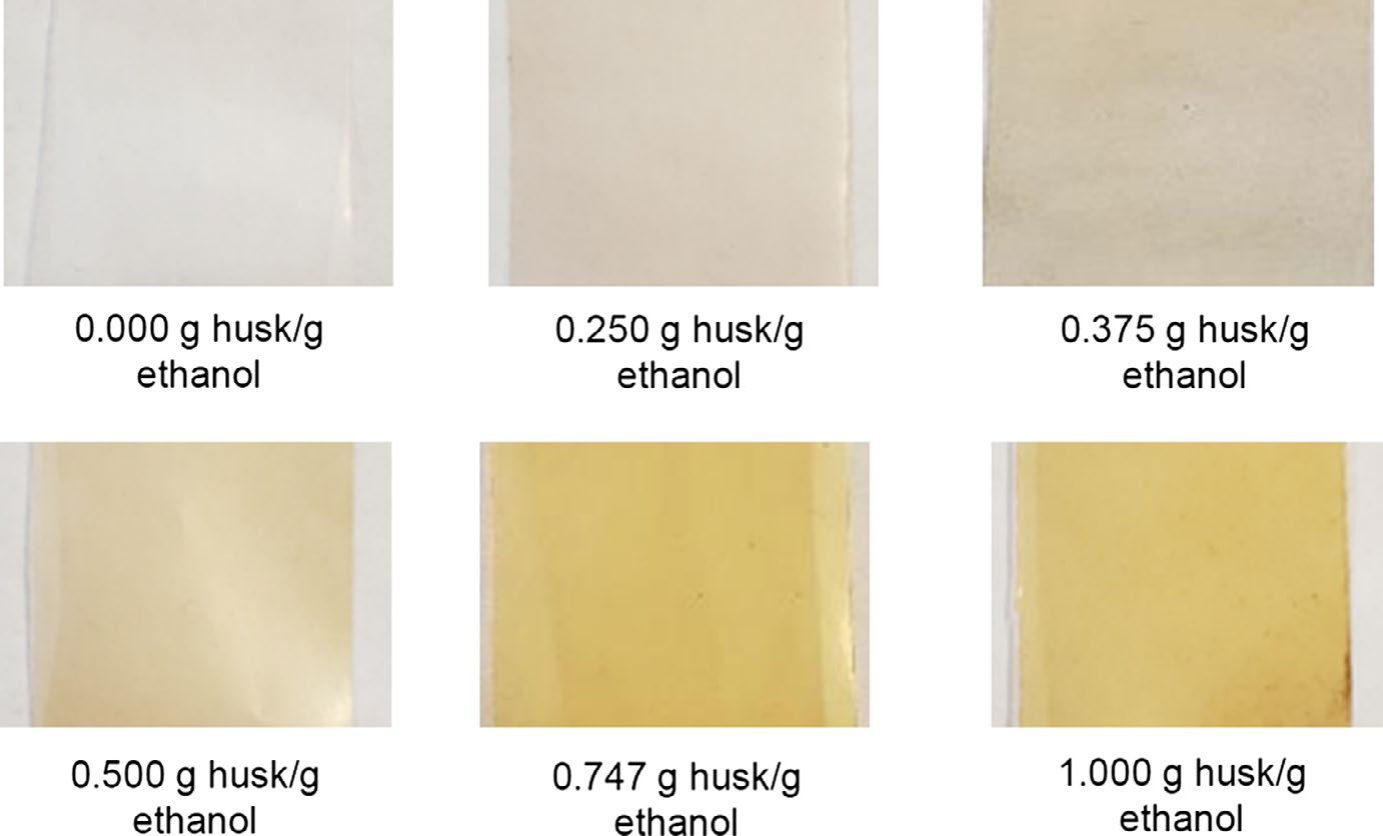
Walnut husk extract impregnated into polymer film color changes as the walnut husk loading is increased

Upon removal from the pressure vessel, the treated, impregnated films were photographed in similar lighting, distance, and background, Figure 3. When compared to the control film that had been exposed for 60 min to the extraction solvent with no added walnut husk, the films became increasingly more yellow as the walnut husk load increased. This color change also corresponds to films increasing mass due to impregnation. Additionally, the mass of the films was analyzed over 50 days exposed to a variety of conditions: stored in a cold, dark nitrogen‐purged container, stored in a cold, dark, nitrogen‐purged container though wiped with a tissue before measurement, stored in a cold, dark, container open to air, immersed in water, and exposed to air and daylight. It was found that there was a negligible change of mass at all storage conditions during that time, which is indicative that the impregnated material is stable within the film or the rate of diffusion out of the films is inconsequential over that time.

### 3.2 FTIR analysis

**Figure 4:**
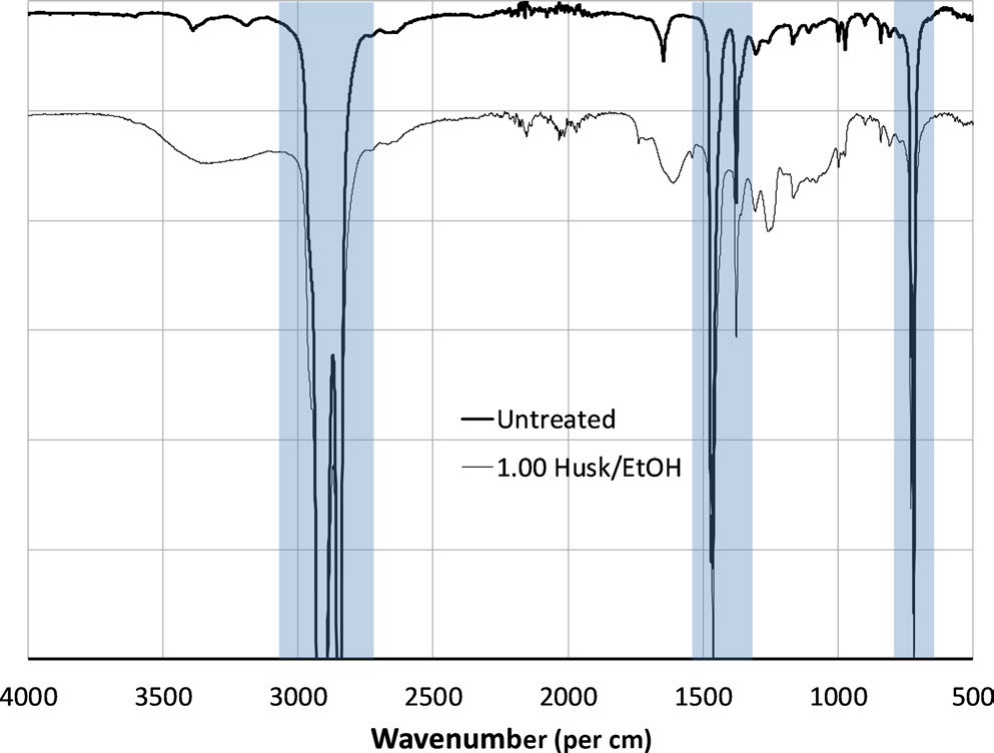
FTIR spectra of untreated poly(ethylene) film (thick line) and polyethylene film impregnated with wlanut husk extract (thin line). The spectrum for the impregnated film is offset by 10%. Polyethylene peaks are highlighted. The walnut husk‐impregnated film was produced using supercritical carbon dioxide with an ethanol modifier and a walnut husk to ethanol mass ratio of 1.00

**Figure 5:**
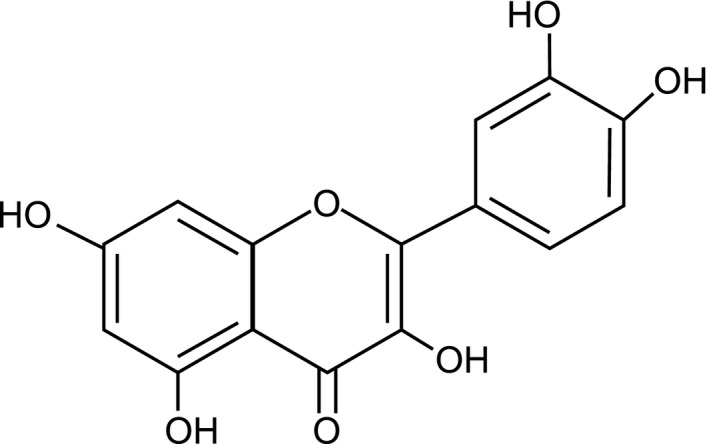
Structure of quercetin that shows the presence of common functional groups of phenolic antioxidants: O‐H, C=O, C‐O, and the aromatic rings

The walnut husk‐impregnated films were analyzed by Fourier Transformed Infrared Spectrometry (FTIR) to determine functional groups present in the compounds that were impregnated into the film. Figure 4 shows the FTIR spectra of the untreated film and an impregnated film. Besides the peaks due to the polyethylene film, the impregnated film has a broad band between 3,650 and 3,000 cm^−1^, which corresponds to the stretching vibrations of the phenolic O‐H groups in the antioxidants (Abdollahi, Taghizadeh, & Savani, 2018). In addition, there are overlapping peaks in the region of 1,750–600 cm^−1^, which are likely due to the stretching vibrations of the carbonyl group (C=O) commonly found in the region of 1,750 cm^−1^–1,650 cm^−1^ and the C‐O bond commonly found in the region of 1,150 cm^−1^–1,050 cm^−1^, as well as the stretching vibrations of the C=C bonds in the aromatic ring commonly found at 1,500 cm^−1^ and 1,600 cm^−1^ and the bending vibrations of the hydrogen atoms in the aromatic ring (commonly found in the region of 800 cm^−1^–600 cm^−1^). These are functional groups found in common phenolic antioxidants such as quercetin, whose structure is depicted in Figure 5, which is used here as a control for comparison in this study. Similar FTIR peaks were observed in PET/PP films impregnated with antioxidants from olive leaf extracts using supercritical carbon dioxide (Cejudo Bastante et al., 2019). FTIR analysis was used to demonstrate the impregnation of eugenol into LLDPE using supercritical carbon dioxide and impregnation of walnut husk extract into biodegradable polyesters by sulfonation (Goni et al., 2016; Latos & Masek, 2019). Therefore, the result of the FTIR study is consistent with the conclusion that some of the antioxidants from the walnut husks are present in the impregnated film.

### 3.3 Biological relevance testing

**Figure 6:**
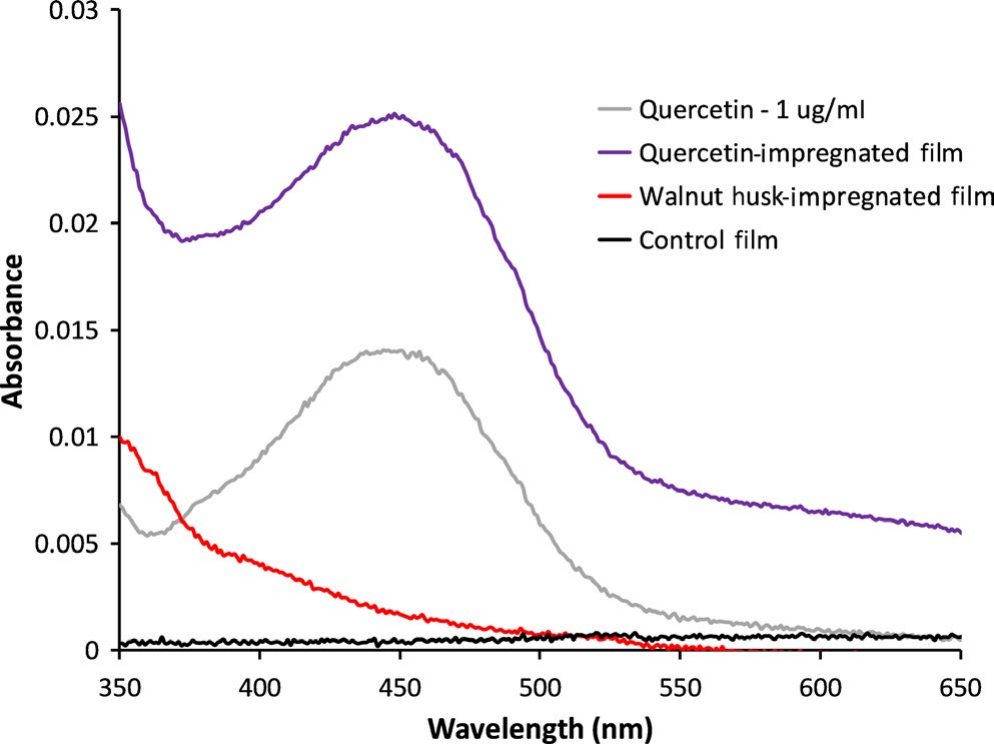
Absorbance spectra of quercetin and the leaching solution of three films. One μg/mL quercetin is shown in gray, a quercetin impregnated film is shown in purple, a walnut husk‐impregnated film is shown in red, and an unimpregnated control film is shown in black

In order to test the potential biological effect of extract impregnation, the film with a mass loading of 1:1 (husk/ethanol) was tested for the ability to leach into the solution and subsequently reduce the ubiquitous heme‐containing protein cytochrome *c*. In addition, film impregnated with quercetin and a nonimpregnated film were also studied. Leaching was detected through the presence of an absorbance below 550 nm in the soaking solutions, which is a hallmark of the presence of flavonoids, the class of common phenolic antioxidants found in plants which includes quercetin (Saltmarsh, Santos‐Buelga, & Williamson, 2003). A standard curve was made for quercetin using the absorbance at 444 nm, yielding the equation (*R* ^2^ = .998 using five data points) Abs_444_ = 0.0179 * [Quercetin_µg/mL_] − 0.0014. This correlation allowed for an estimation of the concentration of quercetin to be 1.47 μg/ml in the leaching solution using a 6 mm circle of film impregnated with quercetin in 350 ml of pH 7.4 buffer. Not only was there an absorbance at 444 nm from the quercetin impregnated film, but the spectra also closely matched that of soluble quercetin as seen in Figure 6, indicating that the impregnation process did not significantly modify the chemical structure of quercetin. The solution soaking the walnut husk‐impregnated film also showed an increased absorbance below 550 nm when compared with a control film (see Figure 6), but its profile did not match that of quercetin, and thus likely contains a more complex mixture of compounds.

**Figure 7:**
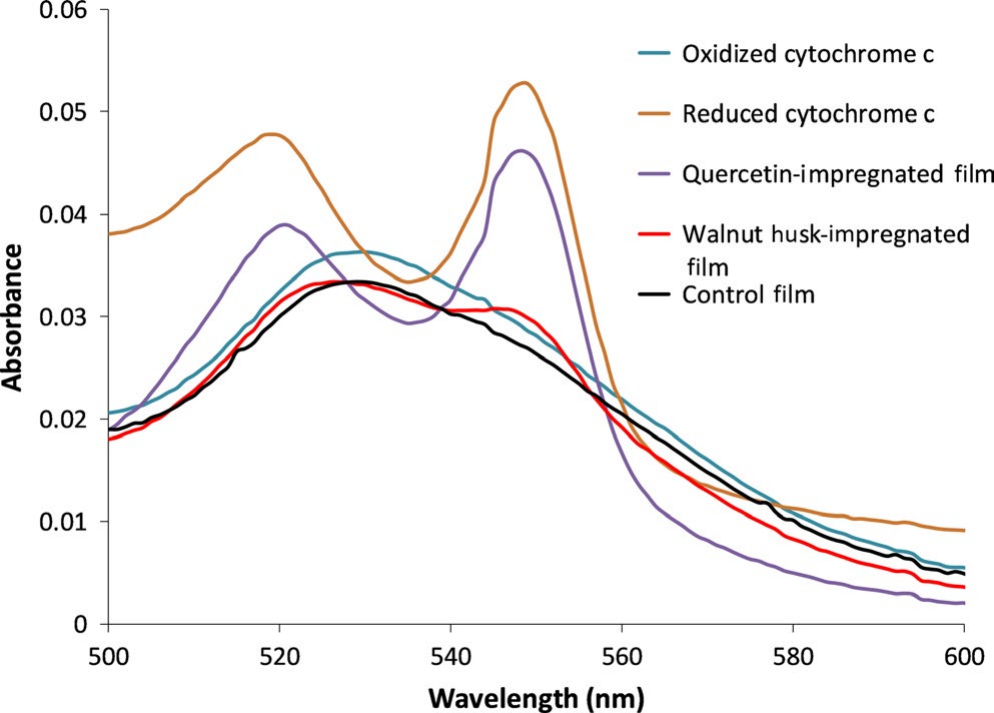
Absorbance spectra of cytochrome *c* after exposure to films for 3 hr. Ability for impregnated films to reduce the protein cytochrome c is tracked via an absorbance peaks at 520 and 550 nm, which corresponds to the signature absorbance maxima seen in the reduced cytochrome c but not the oxidized cytochrome c spectrum. Reduced cytochrome *c* is shown in orange while oxidized cytochrome *c* is shown in blue. A quercetin impregnated film is shown in purple, a walnut husk‐impregnated film is shown in red, and an unimpregnated control film is shown in black

While the above data indicate that flavonoids are able to be transferred from impregnated films, it is important to also know whether they are able to be effective as antioxidants. The ability of these films to reduce proteins was tracked through the appearance of distinct peaks at 520 and 550 nm when the films were incubated (Margoliash & Frohwirt, 1959). Oxidized cytochrome *c* does not produce these distinct peaks and instead has a broader absorbance peak whose maximum is around 530 nm. The quercetin‐impregnated film appeared to almost completely reduce cytochrome *c* after 3 hr, while the walnut husk‐impregnated film still reduced the protein, just to a lesser degree, as seen in Figure 7. The control film with no impregnation shows no ability to reduce cytochrome *c*.

## 4 CONCLUSIONS

Antioxidant compounds were simultaneously extracted from walnut husks and impregnated into LDPEs in the same agitated batch pressure vessel. The simultaneous extraction and impregnation took place under agitation at 60°C, 19.4 MPa, using 15 wt‐% ethanol in supercritical carbon dioxide with a hold time of 60 min. The walnut husk to ethanol mass loading was varied from 0.25 to 1.0, with the highest mass loading resulting in the greatest mass of antioxidant compounds impregnated into the polymer. The presence of antioxidant compounds in the polymer was determined by FTIR as well as the film's ability to reduce the heme‐containing protein cytochrome *c* compared against a control. The film mass remained stable for the duration of a time study lasting 50 days. This work shows that compounds can jointly be extracted from plant matter and impregnated into polymer films in‐situ*,* presenting a potential novel technique for producing active packaging.

## CONFLICT OF INTEREST

The authors declare that they do not have any conflict of interest.

## ETHICAL APPROVAL

This study does not involve any human or animal testing.
